# Advancing Strategic Health Purchasing for Primary Healthcare in Southeast Asia: A Review of Policy and Practice in Indonesia, the Philippines, Thailand, and Vietnam

**DOI:** 10.34172/ijhpm.9326

**Published:** 2026-07-28

**Authors:** Capucine Barcellona, Michael Caampued, Shita Dewi, Jeremy Turner-Gorospe, Theepakorn Jithitikulchai, Nguyen Khanh Phuong, Nguyen Thu Ha, Ryan Rachmad Nugraha, Ong The Due, Gugma Vidal, Pacharee Wattanakulchai, Kiesha Prem

**Affiliations:** ^1^Saw Swee Hock School of Public Health, National University of Singapore, Singapore, Singapore.; ^2^Philippine Society of Public Health Physicians, Manila, Philippines.; ^3^Centre for Health Policy and Management, Faculty of Medicine, Public Health and Nursing, Universitas Gadjah Mada, Yogyakarta, Indonesia.; ^4^Faculty of Economics, Thammasat University, Bangkok, Thailand.; ^5^Health Strategy and Policy Institute, Ministry of Health, Hanoi, Vietnam.; ^6^Department of Health Policy and Economics, Hanoi University of Public Health, Hanoi, Vietnam.; ^7^Department of Family and Community Medicine, Faculty of Medicine, Public Health and Nursing, Universitas Gadjah Mada, Yogyakarta, Indonesia.

**Keywords:** Strategic Purchasing, Primary Healthcare, Universal Health Coverage, Southeast Asia, Health Financing, ASEAN

## Abstract

**Background:**

Strategic health purchasing is a critical lever for advancing universal health coverage (UHC), particularly in low- and middle-income countries (LMICs). Across Southeast Asia, countries are reforming their purchasing functions to enhance the efficiency, equity, and quality of primary healthcare (PHC). This study assesses the extent and progress of strategic purchasing for PHC in four Association of Southeast Asian Nations (ASEAN) member states: Indonesia, the Philippines, Thailand, and Vietnam.

**Methods:**

We applied the Strategic Health Purchasing Progress Tracking Framework to conduct a structured comparative assessment across five purchasing dimensions: financial management, benefits specification, contracting arrangements, provider payment, and performance monitoring. Data was collected through semi-structured interviews with national stakeholders, supplemented by grey literature and policy document reviews. The data was analysed, triangulated and validated through expert consultation and thematic analysis.

**Results:**

All four countries have taken steps toward institutionalising strategic purchasing through national health insurance (NHI) schemes, though the depth and coherence of reforms vary. Thailand’s universal coverage scheme (UCS) demonstrates the most advanced implementation, characterised by performance-based payment mechanisms and relatively strong monitoring systems. Indonesia and the Philippines are implementing pilots to align payments with service delivery goals, though challenges persist in integrating PHC with broader health system functions. Vietnam maintains high insurance coverage but exhibits limited strategic purchasing practices, particularly for PHC. Across all countries, gaps in data systems and governance capacity constrain effective implementation.

**Conclusion:**

Strategic purchasing reforms in Southeast Asia are progressing but remain uneven in scope and institutional maturity. Strengthening policy coherence, investing in health information systems and aligning public financing with PHC priorities will be critical to advancing these reforms. In particular, Thailand’s experience offers relevant lessons for broader regional efforts to operationalise strategic purchasing for more resilient and equitable PHC systems.

## Background

**Key Messages**

**Implications for policy makers**

- Strengthen regional collaboration and knowledge-sharing for strategic purchasing and primary healthcare (PHC) in Southeast Asia, leveraging existing health networks and forums. Promote open discussion on policy enablers and challenges in implementing strategic purchasing, enabling countries to learn from the experiences of their peers.
- Reinforce PHC as the foundation of universal health coverage (UHC) in Southeast Asia by implementing appropriate referral and gatekeeping mechanisms; designing benefit packages centred around primary care; and incentivising primary care utilisation through strategic provider payment mechanisms, contracting and/or financial incentives.
- Invest in the supply-side provision of primary care, including workforce development and supplies of basic medicines—including in rural and hard-to-reach areas.
- Align PHC budget allocations with population health needs and system performance, using data on utilisation, epidemiological profiles, and provider performance to inform resource allocation.
- Reduce fragmentation in health financing schemes to enhance purchasing power, improve efficiency and promote equity in access to primary care benefits across the population.

**Implications for the public**

This research highlights the importance of strategic purchasing—the evidence-based allocation of health funds from a “purchasing agency” to healthcare providers—to improve primary healthcare (PHC) in Southeast Asia. This piece points to the need to not only raise and pool funds for healthcare, but also to allocate them effectively to achieve universal health coverage (UHC). Some key principles of strategic purchasing for PHC include provider performance monitoring and selection; benefit package design rooted in PHC; and incentives to providers that reward good performance and a focus on prevention. Southeast Asian countries are currently experimenting with diverse strategic purchasing models for PHC, and knowledge exchange across countries can facilitate this regional journey. The implications of this research are universal and have relevance for national and provincial health administrators, healthcare provider networks, patients, and members of the public interested in the Association of Southeast Asian Nations (ASEAN) cross-government relations.

The Association of Southeast Asian Nations (ASEAN) member states are currently striving for universal health coverage (UHC), having endorsed the “ASEAN Declaration on Strengthening Social Protection” in 2013 and outlined various national health strategies focused on improving access and health coverage.^1^ The World Health Organization (WHO) defines UHC as universal access to a full range of quality health services without financial barriers, and acknowledges that the path to UHC will be different for every country.^2^ UHC must go beyond political commitments, however, and must be achieved through health systems strengthening and effective resource allocation. This requires well-crafted health financing policies centred around equity and sustainability.

 The WHO identifies three core functions of health financing.^3^ The first is revenue raising, which involves raising money for health through domestic and external sources, including government budgets, out-of-pocket (OOP) payments from households, compulsory health insurance schemes, voluntary health insurance schemes, and donor aid. The second function is fund pooling, which refers to the accumulation of prepaid health funds on behalf of a participating—whether the entire national population or a subset—to spread out the financial risk of paying for health services. These funds could be pooled through insurance contributions, taxation revenue or a mix of both. The third is health purchasing, which is the process of using pooled funds to pay healthcare providers for delivering a defined set of services, known as the benefits package, specified or unspecified. Purchasing involves four core questions: (1) what to buy, (2) from whom to buy, (3) how to buy, (4) at what price.

 These interrelated functions signal that, while raising funds for health is important, this alone will not be enough to achieve UHC. Determining *how *pooledfunds are spent—the purchasing function—is especially critical to low- and middle-income countries (LMICs) with limited capacity to increase their resources and/or facing decreasing availability of donor aid as they shift toward middle-income status.^4-6^

 The evidence-based and effective purchasing of health services—known as strategic purchasing—has emerged as a tool for providing high-quality, equitable and cost-effective care. Strategic purchasing is a model of health purchasing that is based on data-driven decision-making. This process involves multiple relationships between a purchaser (or purchasing agency), healthcare providers and a population entitled to receive preventive and curative care.^7^ Collective purchasing power, performance incentives and other mechanisms can drive high-performing, patient-centred care by incentivising providers to improve the quality of their services.

 Purchasing strategically requires a purchasing agency to use information on people’s health needs, preferences and values, and to then make critical assessments of what health services to buy, which healthcare providers to purchase from and what methods of purchasing to use. As compared to “passive purchasing,” *strategic* purchasing involves a continuous evaluation of how to allocate resources more efficiently. This can potentially clear up fiscal space to fund social protection schemes and target gaps in the healthcare system, resulting in wider access to quality health services for more people.

 As of 2024, ASEAN member states are at different stages of implementing strategic purchasing in their health systems. Some countries, such as Thailand, have become innovators in the field by implementing strong governance, performance monitoring and accountability mechanisms.^7,8^ Other countries are carrying out smaller-scale strategic purchasing pilots; others yet lack concrete plans for implementation but have indicated an interest in developing strategic purchasing.^9^ Despite these different stages of development, there is a growing recognition in Southeast Asia that leveraging strategic purchasing could support UHC. This is particularly relevant to purchasing primary healthcare (PHC)—the major safety net in public health and cornerstone of UHC.^10^ While strategic purchasing is relevant across all levels of care, including specialist and hospital services, this paper focuses specifically on PHC given its centrality to UHC policy in the Southeast Asian region and the distinct purchasing mechanisms it requires.

 This paper outlines health purchasing for PHC in four ASEAN member states at different stages of strategic purchasing implementation: Indonesia, the Philippines, Thailand, and Vietnam. The purchasing practices of these four countries are considered in light of the passive- to strategic- purchasing continuum, mapping out levels of readiness and major purchasing reforms in support of UHC. Through a close analysis of these four countries, this study aims to assess Southeast Asia’s progress in strengthening its PHC systems through strategic purchasing. In doing so, it offers practical insights for policy-makers in LMICs seeking to improve PHC purchasing as a pathway to UHC.

 Southeast Asia has made significant progress toward economic development and improved health outcomes, including reductions in maternal and child mortality, increased life expectancy and growing access to essential health services.^11-14^ Nonetheless, OOP spending remains high, averaging of 35% of total health expenditure in the ASEAN bloc and reaching over 60% in some countries (71% in Myanmar, 61% in Cambodia).^15^ The majority of ASEAN countries face fragmentation across different health financing schemes, posing challenges to cost-effective resource allocation and pooled purchasing power; and, even when large national health insurance (NHI) schemes are in place, these may lack the funds to provide comprehensive benefits packages. Furthermore, COVID-19 further exposed systemic vulnerabilities in regional health systems, with the region’s 218 million informal sector workers particularly at risk and the long-term impact of the pandemic on Southeast Asia’s economy still unknown.^16^ In this context, ASEAN countries must make the best use of limited healthcare funds through deliberate health financing policies and process improvements. A balance must be sought between preventive, primary and tertiary care, as ASEAN health systems remain fragmented and reliant on hospital care.^17^ Though a “South-East Asia regional strategy for PHC: 2022-2030” was developed by the WHO in consultation with regional Health Ministers, some significant implementation roadblocks will need to be overcome.^18,19^

 The four countries represented in this study—Indonesia, the Philippines, Thailand, and Vietnam—have all made deliberate progress toward UHC and health financing reform in recent years. In particular, all four have made great strides in establishing NHI schemes, thus avoiding the health financing scheme fragmentation seen in other Southeast Asian countries. Indonesia, whilst still in the early stages of strategic purchasing implementation, has made significant progress in recent years with the launch of its NHI scheme (Jaminan Kesehatan Nasional, JKN) and the integration of health purchasing activities under BPJS-K (Badan Penyelenggara Jaminan Sosial Kesehatan, the implementer of JKN). In doing so, it has established the world’s largest single-payer health insurance system in the world.^20^ The Philippines promulgated its UHC Law in 2019, and has made great strides in implementation despite the disruption of COVID-19.^21^ A regional and global pioneer of UHC, Thailand is also continuously strengthening its strategic purchasing practices by refining the role of the National Health Security Office (NHSO) and targeting health system gaps through performance monitoring and responsive policy-making.^7^ Finally, Vietnam has expanded its social health insurance (SHI) coverage to over 93% of the population, administered by Vietnam Social Security (VSS). The “National Strategy for the Protection, Care and Improvement of People’s Health by 2030, with a Vision to 2045” was also developed to establish a basic health service package and payment methods prioritising PHC.^22^

## Methods

 This study forms part of a broader multi-country assessment of health purchasing in all ASEAN member states (Brunei Darussalam, Cambodia, Indonesia, Lao PDR, Malaysia, Myanmar, the Philippines, Singapore, Thailand, and Vietnam) conducted by the Southeast Asia Regional Collaborative for Health, a learning network working to raise regional capacity for UHC.

###  Data Collection

 Four countries were selected for in-depth analysis—Indonesia, the Philippines, Thailand, and Vietnam—due to their large population sizes, political influence in the ASEAN bloc and diversity of health system structures. These countries also represent different stages of implementation in strategic purchasing for PHC, and their respective progress toward UHC may hold relevant lessons for other health systems in transition.

 The study relied on two primary sources of data. Firstly, a comprehensive literature review was conducted using search engines and government archives. The sources considered included peer-reviewed studies, government documents, financial reports, legislation, and other grey literature at the regional and national level. Secondly, in-depth semi-structured interviews lasting 30 minutes to 3 hours were conducted with 14 stakeholders across the four countries. Interviews included national and subnational policy-makers, health financing experts, academics, and representatives from purchasing agencies. The interviews were conducted in-person and virtually in English or local languages using a standardised interview guide. Informed verbal consent was obtained in all cases.

###  Data Analysis

 The data on each country was analysed thematically according to the “Strategic Health Purchasing Progress Tracking Framework,” developed by the Strategic Purchasing for Africa Resource Centre to assess countries’ progress across core elements of health purchasing. The framework evaluates country performance across five dimensions: financial management, benefits specification, contracting arrangements, provider payment, and performance monitoring (Table 1).^23,24^

**Table 1:** Dimensions of the Strategic Purchasing Progress Tracking Framework

**Dimension**	**Definition**
Financial management	“The purchaser’s ability to project and manage revenue and expenditures. The purchaser needs to know how much money is available to provide the benefit package and to pay providers for contracted services.”
Benefits specification	“This includes selecting the services and interventions to be included in the benefit package, the service delivery standards, where and how the services can be accessed (including gatekeeping policies), how much of the cost of services will be covered by the purchaser (and accompanying cost-sharing policies), and which medicines will be covered.”
Contracting arrangements	“This includes systems and policies for selecting public and/or private providers to deliver services in the benefit package, entering into contracts with them that specify terms and conditions (eg, at which level specific services can be delivered and data reporting requirements), and enforcing the contracts.”
Provider payment	“This includes systems and policies for selecting, designing, and implementing provider payment systems and setting payment rates.”
Performance monitoring	“This includes systems and processes for assessing provider performance, providing feedback for improvement, and carrying out system-level analysis of utilization, quality, and so forth to inform purchasing decisions.”

 Data from the literature review and interviews were triangulated across multiple sources. The data was deductively coded and categorised according the Framework above by SD and RRN (Indonesia), MC and JTG (the Philippines), TJ and PW (Thailand) and NKP, NTH, and OTD (Vietnam). The coding was cross-checked and validated by CB and KP. All country-level profiles were analysed and reviewed by at least two researchers for accuracy and consistency.When considering the health purchasing practices in our four countries along the passive to strategic purchasing continuum, attention was paid to the characteristics of these two modes of purchasing as described in Table 2. Importantly, as purchasing lies on a spectrum from wholly passive to strategic, countries were often placed along this spectrum rather than in one category exclusively.

**Table 2:** Characteristics of Passive Versus Strategic Purchasing

**Element**	**Passive Purchasing **	**Strategic Purchasing **
Governance – Roles and responsibilities	Fragmentation, poor coordination and unclear division of responsibility or oversight between health system actors (eg, MoH, purchasing agency) over decision-making.	Clear roles and responsibilities are set for different health system actors.
Governance – Purchaser mandate and autonomy	Purchasing agency lacks a strong mandate and has no to limited autonomy over purchasing decisions.	Purchasing agency is given the mandate to carry out *strategic* purchasing through sufficient budget and decision-making autonomy.
Governance – Accountability mechanisms	No clear lines of accountability in the implementation of purchasing policies.	Arrangements for accountability are made and monitored. This may involve patient organisations, expert groups, financial oversight institutions, quality assurance mechanisms, or accreditation agencies.
Public financial management	Broader public financial management rules are rigid and input-based.	Public financial management rules allow for strategic purchasing to be implemented through diverse payment methods, such as outcomes-based or performance-based payments.
Purchasing power	The purchasing agency’s purchasing power is limited by factors such as the small size of pooled funds, multiple fragmented health financing schemes, a lack of coherence across schemes or a limited mandate for the purchaser to implement strategic purchasing, such as in tax-funded health systems.	The purchaser has sufficient purchasing power through an adequately-sized fund pool and low scheme fragmentation, allowing it to influence provider performance and health service delivery.
Budget	Providers receive funds/budget allocations automatically or are paid through input-based mechanisms (fee-for-service), with no consideration of performance.	The transfer of funds is linked to evidence on the performance of providers, population health needs and other relevant metrics.
Responsiveness	Purchasing decisions are rigid and not responsive to changes in population health needs, local circumstances and other factors.	Purchasing decisions are responsive to the local context, and any changes required can be implemented within reasonable time frames.
Performance monitoring	Absence of regular performance monitoring on individual providers and/or the wider health system. Any limited performance monitoring functions do not influence purchasing decisions.	Provider and health system performance is monitored in a regular, structured fashion. The information is used to shape future purchasing decisions.
Information management systems	Information management systems may be inappropriate, out of date and/or paper-based, preventing high-quality data collection and meaningful performance monitoring. Information that is collected is not routinely used for decision-making on strategic purchasing and provider payment.	Digitalisation and health information management systems enable adequate data collection and analysis. This facilitates evidence-based purchasing mechanisms like pay-for-performance.
Purchaser behaviour	The purchaser is unable to influence how health services are provided due to limited capacity, know-how, purchasing power or other.	The purchaser effectively influences provider behaviour, including the quantity and quality of health services offered to a population.
Transparency	Low transparency on how health purchasing and financing decisions are made.	High transparency over purchaser and provider decisions for the population being served.
Benefits packages	No clear benefits package in place, leading to confusion among purchasing agencies and providers; alternatively, the benefits package may not be appropriately implemented, not regularly revised or not set and implemented strategically based on population health needs.	An explicit benefits package exists; purchasers know what to purchase and providers know what to provide, including cost-sharing arrangements and gatekeeping mechanisms.
Benefits package revisions	Irregular revision of the benefits package and no structured mechanism to make changes.	Clear processes and timelines for the revision of benefits packages, inclusive of Health Technology Assessments and civil society participation.
Service delivery	Poor purchasing decisions or payment in reimbursement prevent providers from delivering the services outlined in the benefits package.	Purchasing decisions and timelines empower providers to deliver all services guaranteed in the benefits package.
Provider incentives	Provider incentives do not reward good behaviour; providers are incentivised to over-or under-treat patients or deliver less impactful interventions.	Providers are incentivised to deliver integrated, high-impact and high-quality care, and rewarded for doing so through appropriate payment methods and incentives.
Performance-based financing	No performance-based financing pilots or initiatives exist.	Performance-based payment mechanisms are integrated within the health system as appropriate, with proper means of verification and fund disbursement.

Abbreviation: MoH, Ministry of Health.

## Results

 Between 2000 and 2021, the UHC service coverage index—an index combining 14 tracer indicators of service coverage into a single summary measure for Sustainable Development Goal (SDG) Indicator 3.8.1—showed improvements in UHC across all four countries analysed in this study, as well as in the broader Southeast Asia region and globally (Figure).^25^ The four countries studied have been successful in establishing compulsory, large-scale health insurance schemes. In the case of Indonesia, the Philippines, and Vietnam, there is one primary social health protection scheme and national purchaser of health services; in the case of Thailand, there are three major health financing schemes with three different purchasers—yet these schemes are non-competing, compulsory and harmonised to some extent. In each country, financing for curative primary care is primarily channelled through these large schemes. Supply-side financing is also provided from national, regional and/or local governments for preventive, promotive and population-level care, and to offer additional support for public sector primary care facilities (eg, facilities maintenance).

**Figure:**
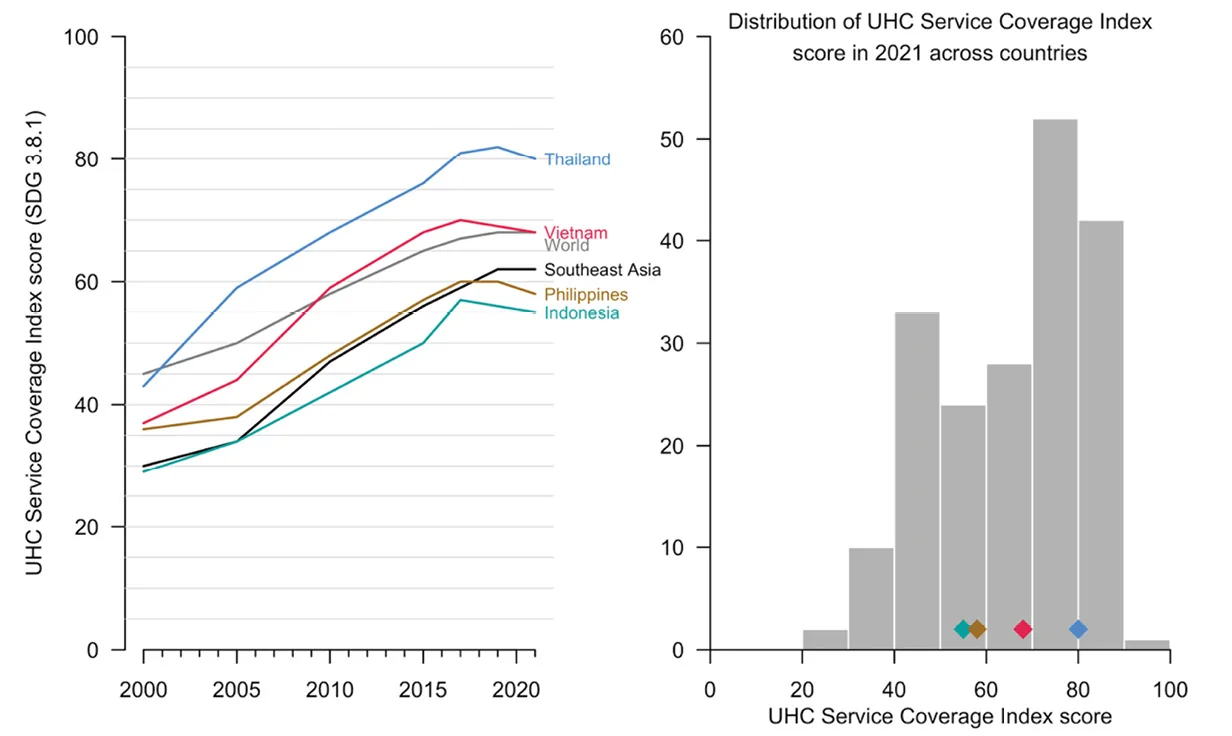
Trends in Universal Health Coverage Service Coverage Index (SDG 3.8.1) Scores From 2000 to 2021 for Four Southeast Asian Countries, Compared With Regional and Global Averages. The left panel shows progress in UHC service coverage (SDG 3.8.1) for Thailand, Vietnam, the Philippines, and Indonesia over two decades. Regional (Southeast Asia) and global (World) averages are included for context. The distribution of UHC service coverage index scores across countries worldwide in 2021, with coloured points indicating the position of each of the selected four Southeast Asian countries, is presented in the right panel. Abbreviations: SDG, Sustainable Development Goal; UHC, universal health coverage.

 The overview for each country along the Strategic Purchasing Progress Tracking Framework is presented below. Full findings are provided in Supplementary files 1-4.

###  Indonesia

####  Background

 Indonesia is an upper-middle income country of 275.5 million people whose historically fragmented health financing system was consolidated in 2014 under the JKN single-payer scheme—now the world’s largest, covering over 98% of the population.^26^ However, its archipelagic geography, decentralised governance, supply-side limitations, and variable district-level allocations for health continue to create structural barriers to effective health financing integration and exacerbate urban-rural inequalities.^27-30^

####  Primary Healthcare System

 Public PHC is delivered through over 10 000 community health centres (Puskesmas) and village-based services (Posyandu/Poskesdes), which provide both curative and population-level preventive care. Curative care is funded through the JKN and delivered at Puskesmasas well as contracted private clinics.

####  Financial Management

 BPJS-K, the fund manager for JKN, pools contributions and government subsidies into a single fund known as Dana Jaminan Sosial. Public facilities receive additional supply-side central and regional government funding, although this investment may not always match local needs. There are ongoing challenges relating to the JKN’s financial sustainability, with a “missing middle” of informal workers struggling to maintain active membership and contributing to a persistent cumulative deficit.^31^

####  Benefits Specification

 The JKN defines a comprehensive primary care benefits package—including consultations, diagnostics, medications, immunisation, screening, and teleconsultation—developed by the Ministry of Health (MoH) through cost-effectiveness assessments and regularly revised through Presidential Regulations and Amendments.^32^

####  Contracting Arrangements

 Puskesmas and private clinics must undergo BPJS-K credentialing and MoH-supervised accreditation to participate in the JKN. The absence of formal contracts for public providers limits strategic purchasing capabilities, and has contributed to poor quality monitoring and facility overcrowding—diminishing patient satisfaction and delaying essential treatments.^33-36^ Consequently, while the automatic involvement of public providers ensures a baseline of access, it reinforces a passive purchasing model that struggles to reward high-performing facilities or address localised system bottlenecks.

####  Provider Payment

 PHC providers receive monthly capitation payments, with rates determined by the MoH. Currently, capitation rates are higher for private than for public providers and are widely considered insufficient.^31,37^ Complex claims administration and procedures that exceed capitation caps have been found to distort provider behaviour, often incentivising unnecessary referrals to higher levels of care.^38^ Performance-based adjustments were introduced in 2019 based on contact rates, chronic disease management and referral ratios.^39^ Additional incentives are available based on claims from PHC centres offering inpatient services, such as birth deliveries.^40^ This performance framework, however, risks penalising under-resourced rural facilities.^41^ Beyond the JKN, public providers receive an additional operating budget from the MoH on an annual basis.

####  Performance Monitoring

 Provider performance is tracked through the BPJS-K’s P-Care information system application. A national reporting platform (ASDK, Aplikasi Satu Data Kesehatan — Health Single Data Application) gathers performance indicators on Puskesmas. Quality and cost control committees (Tim Kendali Mutu dan Kendali Biaya — Cost and Quality Control Committee) are based at the national, provincial and BPJSK branch-levels to monitor health service quality. They conduct ad-hoc utilisation and referral ratio reviews for PHC, though the extent to which this determines purchasing decisions is unknown. Overall, fragmented data systems and inconsistent implementation across provinces limit the practice of strategic purchasing.

###  The Philippines 

####  Background

 The Philippines is a lower-middle income archipelagic country of 115.56 million. Its 2019 UHC Act catalysed system-wide reforms,^42^ with near-universal coverage under the national PhilHealth insurance scheme alongside devolved local government units (LGUs) responsible for supply-side PHC delivery for 18 800 to 4 345 000 inhabitants.

####  Primary Healthcare System

 The Local Government Code of 1991 charges LGUs to manage rural health units and barangay health stations to deliver PHC services.^43^ Rural health units receive LGU and central Department of Health (DoH) funding. This is supplemented by PhilHealth’s Konsulta programme for PHC, contracting both public and private facilities. However, widespread bypassing of PHC in favour of hospital care continues to undermine system efficiency.

####  Financial Management

 Duplicative allocation streams exist within the Philippine health financing systems. LGU-level funds are aggregated through various sources, including Internal Revenue Allotment local revenues, DoH funds, PhilHealth payments, grants, and donor loans.^44^ LGUs therefore have devolved functions for health but also receive DoH funding for supply-side readiness. Efforts to devolve more health financing responsibilities from the DoH to LGUs have been made through the Mandanas-Garcia ruling and DoH Devolution Transition Plan 2022-2024, but these remain incomplete.^45,46^ While PhilHealth enjoys relatively greater flexibility in financial management, its activities are largely directed toward specialist care rather than PHC.

####  Benefits Specification

 LGUs do not define explicit benefits packages, but are mandated to deliver population-based PHC under the Local Government Code and UHC Law. PhilHealth’s Konsulta package defines a specific set of consultations, screenings, diagnostics, and medicines now available free at the point of care to all Filipinos.

####  Contracting Arrangements

 LGU PHC networks borrow their accreditation standards from PhilHealth, and occasionally contract private providers (generally for laboratory works and diagnostics). PhilHealth’s Konsulta package contracts non-hospital public and private facilities through local health insurance offices. However—in spite of global evidence that accreditation improves care standards—the Philippines has faced gaps in inspection capacity, weak mechanisms for the de-accreditation and workforce and financing issues.^47^ Moreover, complex administrative and legal requirements for contracting have limited the private sector’s participation in the implementation of UHC reforms.^48^

####  Provider Payment

 Public PHC providers receive annual line-item budgets from LGUs. Konsulta providers receive advance annual capitation payments at a standardised rate of PHP1700 (~US $30) per capita (a significant increase from earlier rates of PHP500 for public facilities and PHP750 for private facilities).^49,50^

####  Performance Monitoring

 LGUs are monitored by the DoH through a scorecard and are required to conduct annual audits of their networks—though the extent to which these are carried out is unclear. PhilHealth oversees its Konsulta delivery through regular surveillance and monitoring. In 2023, PhilHealth implemented network contracting arrangements to test integrative local health system reforms through the Primary Care Provider Network Sandbox.^51^ The Sandbox involved regular monitoring and evaluation, and led to the rollout of a more expansive network contracting and benefit delivery model termed “Healthcare Provider Networks” in 49 LGUs.

###  Thailand

####  Background

 Thailand is an upper-middle income country of 71.58 million recognised globally for its UHC achievements. It operates three complementary health financing schemes, each with distinct arrangements and managing agencies: the universal coverage scheme (UCS), covering 74% of the population, financed through a tax-based government budget and managed by the NHSO; the Social Security Scheme (SSS) for private employees, managed by the Social Security Office (SSO); and the Civil Servant Medical Benefit Scheme (CSMBS) for civil servants and their families, managed by the Comptroller General’s Department (CGD).^52^

####  Primary Healthcare System

 PHC is delivered through a nationwide network of public health centres and community hospitals (district health systems) under the Ministry of Public Health. PHC in Thailand is supported by strong community health volunteer programmes and mandatory referral pathways.

####  Financial Management

 The NHSO manages UCS funds autonomously following annual parliamentary approval processes. The SSO, under the Ministry of Labour, oversees SSS contributions and manages funds. The CGD oversees CSMBS budget formulation and execution, though it does not have a formal legal mandate to function as purchaser for the scheme.

####  Benefits Specification

 All schemes offer comprehensive PHC benefits packages. These are defined through in the National Health Security Act of 2002 for the UCS, the Social Security Act of 1990 and a technical advisory committee for the CSMBS.^53,54^ Benefits packages are updated periodically, with the NHSO using Health Technology Assessment through the Health Intervention and Technology Assessment Program Foundation programme.^55^ The NHSO also oversees promotive and preventive care for all Thai citizens regardless of scheme membership.

####  Contracting Arrangements

 The NHSO contracts public (mainly district health systems) and private PHC providers through contracting units for primary care, with public providers automatically enrolled and private providers subject to accreditation standards. The SSS contracts large hospitals which can then subcontract PHC clinics. The CGD does not contract providers directly, but reimburses claims against predetermined price ceilings.

####  Provider Payment

 The UCS and SSS use capitation-based payments for PHC, with the UCS incorporating a quality and outcome framework (QOF) for provider performance incentives. The CSMBS reimburses providers on a fee-for-service basis—contributing to per capita expenditure nearly three times that of the UCS and ongoing cost escalation.

####  Performance Monitoring

 The NHSO conducts systematic performance monitoring for the UCS, using microdata across multiple PHC indicators to guide purchasing decisions. The SSO and especially, CGD lack comparable accountability frameworks and performance monitoring functions for PHC.

###  Vietnam 

####  Background

 Vietnam is a lower-middle income country of 98.9 million. Over 93% of the population is covered by the SHI Fund, but OOP payments remain high and financial sustainability is challenged by an ageing population and rising burden of non-communicable diseases.^56,57^ SHI revenues are pooled through various sources, including individual households, employers and government subsidies. The fund holder for the SHI is VSS, though the MoH is responsible for key purchasing and policy decisions. Curative services are provided through the SHI and preventive care is financed by supply-side government budgets.

####  Primary Healthcare System

 PHC has historically been delivered through approximately 11,100 public commune health centres (CHCs), though a major 2025 administrative reform has reduced these to 3321.^58,59^ PHC is also available at higher-level district health centres (DHCs) and private facilities. Though PHC is generally free or low-cost, there is widespread bypassing of CHCs in favour of higher-level care remains—in part, due to Vietnam’s bypassing policy allowing patients to seek treatment without referrals from CHCs.^60^

####  Financial Management

 The VSS operates a single fund, pooling revenues raised at the provincial level. Provincial bodies (SSOs, Departments of Health and People’s Committees) are jointly responsible for local SHI implementation. Preventive services sit outside the SHI, and are annually planned and financed through government budget channels.

####  Benefits Specification

 The SHI defines an explicit curative benefits package for PHC comprising 76 basic services and 241 essential medicines, with co-payments ranging between 0–20%.^61,62^ Preventive services are guided by a MoH Circular No. 30 of 2024, but depend on variable provincial budget allocations for implementation.^63^

####  Contracting Arrangements

 Public facilities directly receive supply-side government funding without formal contracting; nonetheless, public PHC providers delivering SHI-funded services require annual VSS contracts. These are signed the higher administrative DHC level on behalf of subordinate CHCs, which cannot contract directly. The SHI also contracts private clinics, though accreditation and contracting processes remain poorly-defined.^5^

####  Provider Payment

 Preventive care is funded through global budget and line-item government subsidies. Curative SHI care is reimbursed predominantly on a fee-for-service basis, with capitation also used between 2009–2021. Attempts to re-introduce capitation payments in 2021 were abandoned that same year due to inadequate funding formulas disrupted by COVID-19.^64,65^ A high-level process to develop DRG-based payment was initiated in March 2026, but fee-for-service remains the prevalent payment method due to its relative simplicity.

####  Performance Monitoring

 The MoH publishes system-wide indicators that include some PHC indicators—both on curative care (through the SHI) and on priority public health programmes. CHCs report on these measures upward through their higher-level DHCs, which share aggregate reports to the MoH. However, this data is not systematically used to inform purchasing decisions.

Table 3 compares the PHC purchasing arrangements across the four ASEAN member states, focusing on the institutional purchaser, benefits package specification, provider payment mechanisms, contracting arrangements, performance monitoring, and overall strategic purchasing readiness. The table highlights the varied stages of implementation and institutional maturity across countries, illustrating both progress and ongoing gaps in advancing toward more strategic, data-driven, and accountable purchasing systems to support UHC.

**Table 3:** Comparative Summary of Strategic Purchasing for Primary Healthcare in Four Southeast Asian Countries

**Country**	**UHC Score**^a^	**% Of OOP Spending**	**Purchaser**^b^	**PHC Benefits Specified**^c^	**Provider Payment Method**^d^	**Contracting Mechanism**^e^	**Performance Monitoring**^f^	**Strategic Purchasing Readiness**^g^
Indonesia	55	32.96	BPJS-K	Defined by MoH	Capitation and performance incentives	Credentialing via BPJS-K	P-Care app monitoring of providers; independent cost and quality control committees (TKMBK); integrated digital reporting platforms in place	Early stage, expanding performance links
Philippines	58	44.36	PhilHealth LGUs	Via Konsulta (partially defined)	Capitation (Konsulta) and input-based (LGUs)	Accreditation with PhilHealth; Contracts at the LGU level	LGU scorecards; limited PhilHealth systems	Fragmented, growing through Konsulta
Thailand	80	9.21	NHSO–UCS SSO–SSS CGD–CSMBS	Defined and updated by NHSO and followed by the SSS and CSMBS	Blended capitation and pay-for-performance/quality outcomes framework (UCS); Blended capitation, diagnostic related groups and fee-for-service (SSS); Fee-for-service for CSMBS	Direct contracts (public and private) under UCS and SSS	Robust data use by NHSO; limited SSO and CGD monitoring	Advanced (UCS); other schemes relatively lagging, especially the CSMBS
Vietnam	68	39.10	VSS	Circular 39 (SHI-covered curative care); DHCs and CHCs mandated to provide preventive services	Fee-for-service; capitation piloted but discontinued	Contracting with VSS for public providers (DHC-level); private contracting also allowed	Data collected but not used for purchasing	Limited–passive purchasing dominant

Abbreviations: UHC, universal health coverage; OOP, out-of-pocket; BPJS-K, Badan Penyelenggara Jaminan Sosial Kesehatan; MoH, Ministry of Health; TKMKB, Tim Kendali Mutu dan Kendali Biaya; LGU, local government unit; NHSO, National Health Security Office; UCS, Universal Coverage Scheme; SSO, Social Security Office; SSS, Social Security Scheme; CGD, Comptroller General’s Department; VSS, Vietnam Social Security; CSMBS, Civil Servant Medical Benefit Scheme; DHC, district health centre; SHI, social health insurance. ^a^UHC Score represents to the country’s UHC Service Coverage Index score, referring to its ability to provide essential health services to the population. ^b^Purchaser refers to the main institution(s) responsible for financing and contracting PHC services. ^c^PHC Benefits Specified indicates whether countries have explicitly defined the scope of primary care services included in their NHI or public delivery packages. ^d^Provider Payment Method reflects dominant mechanisms for reimbursing primary care providers, with attention to performance-based adjustments or pilot schemes. ^e^Contracting Mechanism describes how providers are accredited or enrolled in purchasing arrangements, including public–private participation. ^f^Performance Monitoring refers to systems in place for assessing PHC service quality and outcomes, and the extent to which this information influences purchasing decisions. ^g^Strategic Purchasing Readiness is a synthesised assessment based on evidence of purchasing autonomy, use of data for decision-making, incentive alignment, and system integration.

## Discussion

 Despite differences in context, all four countries studied—Indonesia, the Philippines, Thailand, and Vietnam—have made meaningful progress toward strategic purchasing for PHC. Several key commonalities can be identified when assessing health purchasing practices specific to PHC.

 One key theme is the role of government budgets in subsidising NHI schemes and/or public health facility operating costs. This is particularly significant to Southeast Asia, where many NHI beneficiaries are unable to meaningfully contribute to premiums and where supply-side readiness for PHC clinics is a challenge in several countries. This trend indicates that, whilst large health insurance schemes have an important role to play in purchasing PHC services, the need for government budgets to implement strategic purchasing should not be overlooked. In the past, strategic purchasing has typically been characterised as requiring a system whereby payers are organisationally separate from healthcare providers (purchaser-provider split).^66,67^ This has been recommended to foster provider competition (and therefore, higher quality) and to enhance accountability: purchasers are enabled to select the best providers and set incentives, such as paying for health outcomes rather than outputs. A purchaser-provider split also clearly defines roles, allowing providers to focus on service delivery and purchasers on operation planning and responding to population needs.^68^ Nonetheless, the institution of a purchaser-provider split in LMICs has not always translated into strategic purchasing.^69^ Experiences from LMICs suggest that strategic purchasing can equally be implemented through government spending, provided that mechanisms for accountability and provider performance management are in place. The role of strategic government purchasing is advocated by Ssennyonjo et al for Sub-Saharan Africa.^70^ The authors describe various mechanisms to make government purchasing more strategic, including aligning public financial management rules with health system objectives; identifying priority populations and health services to direct resources to; taking a multisectoral approach that considers social determinants of health, promotion and prevention; and defining appropriate levels of provider autonomy. Similar considerations apply in Southeast Asia, where NHI schemes are generally not sufficient to cover most healthcare expenses and where government budgets can stabilise fluctuations in NHI budgets. In Indonesia, for example, the JKN scheme suffered a budget deficit in its early years; whilst this was reversed in 2019, the scheme is in a deficit state once more as of 2025. Encouraging strategic resource allocation from government budgets can redirect funds to priority areas, such as HIV/AIDS and tuberculosis, outside of this scheme.

 A further central theme to emerge from our study is countries’ focus on moving toward capitationand, where possible, performance-based payments through pilot initiatives. Piloting performance-based Konsulta payments in 7 provincesis allowing the Philippines to identify successful implementation models before developing broader PHC packages for all. Similarly, strategic purchasing pilots in Indonesia currently include performance incentives for maternal and newborn health services at the primary level. Regular monitoring & evaluation of these pilots is providing crucial information that may guide the JKN in eventual full implementation.^71^ In spite of this growing interest in performance incentives, the international evidence on pay-for-performance is mixed, and implementation characteristics are a key determinant of success. Lessons for the region may be drawn from Thailand’s initial experience of implementing the QOF. Initial challenges were faced, including limited stakeholder engagement and information technology barriers^72^; yet through continuous engagement and refinement, the QOF has remained a promising intervention within Thailand’s broader provider payment landscape.^73^

 Similarly, while countries are generally trending toward capitation payments for primary care, lessons can be drawn from countries’ past experiences. In order to succeed, capitation mechanisms in PHC must be carefully implemented and designed, includingthrough adequate payment timelines and well-calculated formulas that avoid disincentivising quality care. An example of a well-designed capitation formula is that of Thailand’s NHSO, which is age-adjusted and recalibrated annually based on unit cost studies submitted by the NHSO.^74^ While capitation is an important payment mechanism, a good and evidence-based design is crucial to avoid pitfalls such as Vietnam’s abandonment of a novel capitation scheme in 2021 after only 5 months of implementation. Vietnam’s scheme was abandoned primarily because the formula failed to adequately cover healthcare costs for facilities under the SHI scheme, eroding confidence and leading to rapid discontinuation. This contrast between Thailand’s long-standing capitation model and Vietnam’s discontinuation underscores how formula design and regular revision are fundamental prerequisites for capitation to function effectively. As other countries in Southeast Asia look to capitation and/or performance-based payments, the experiences of neighbouring countries can therefore act as illustrative cases. Cross-border cooperation, capacity-building and policy engagement is recommended to allow countries to learn from one another’s models and identify the provider payment mechanisms best suited to their context.

 Finally, a key reflection from our study is the potential for strategic purchasing to contribute to overall health system development and UHC. Since 2000, Asian health systems have made vast improvements to healthcare quality, service coverage and health outcomes such as life expectancy and child survival.^12,13^ In particular, since 2000, maternal and under-5 mortality rates have declined faster in Southeast Asia than in any other WHO region.^75^ These improvements are vastly attributable to UHC plans and policies implemented across the region.^76^ The value of strategic purchasing to support UHC is best evidenced by Thailand, as known pioneer of UHC.^7^ Strategic purchasing has been a cornerstone of Thailand’s UCS since 2002. It benefits from a commitment to continuous improvement from the NHSO and has been instrumental in enabling Thailand’s improvement in the UHC Service Coverage Index (Figure). Indeed, whilst all four countries in this study have advanced toward UHC since 2000, Thailand has seen the greatest UHC Index score increase. Regional progress has unfortunately stagnated in recent years due to financial constraints, supply-side limitations, ageing populations and low levels of public spending, exacerbated by the COVID-19 pandemic and subsequent economic shocks.^11,77^ Learning from Thailand’s decades-long experience of embedding UHC in national policy and strengthening the health system through robust governance and accountability mechanisms, strategic purchasing should be recognised as a critical tool in Southeast Asia—one that could help renew the regional momentum toward UHC.

 Importantly, strategic purchasing is not an end itself; policy reforms must have staying power and direction within a country’s healthcare system in order to pave a path for UHC in the long run. Underlying any effort toward strategic purchasing should be effective governance structures that grant adequate levels of accountability and autonomy to purchasers. This has been largely achieved in Indonesia and Thailand, where autonomous purchasing agencies exist within clear regulatory frameworks on their roles and responsibilities (in Indonesia, Law No. 40/2004 on the Social Security System and Law No. 24/2011 on the BPJS; in Thailand, the National Health Security Act B.E. 2545 of 2002 and the establishment of the National Health Security Board which governs UCS policies and decision-making). The JKN in Indonesia and the NHSO in Thailand are given the mandate to purchase curative care for the vast majority of the population—with rapid progress in Indonesia to cover over 98% of the population—and hold sufficient purchasing power due to the centralisation of previously fragmented schemes. Progress toward the same goal is also visible in the Philippines and Vietnam. The Philippines’ 2019 UHC Law gave all citizens the right to NHI through PhilHealth; in recent years the agency has enhanced its benefits package, refined guidelines for contracting healthcare provider networks and highlighted steps to strengthen governance mechanisms in its 2024 Board Assessment report.^82,83^ In Vietnam, an administrative reform aims to streamline bureaucracy and Resolution 72-NQ/TW was passed in 2025 outlining key measures to improve the healthcare system, aiming for all citizens to receive basic medical care free of charge by 2030.^84^

 Our study has identified several challenges related to the purchasing of PHC services. As ASEAN health systems are centred on specialist care, great efforts must be undertaken to encourage PHC care-seeking through gatekeeping functions, financial incentives and improved care quality, among other methods. One key element will include expanding, and making explicit, benefits packaged centred on primary care. The Philippines, especially, sees less uptake of PHC services among their populations—perhaps due to historical concerns about quality of care and a lack of strong referral systems. If UHC targets are to be achieved by 2030, relevant national insurers’ benefits packages (ie, PhilHealth’s NHI and VSS’ SHI) should shift to place equal, if not greater, weight on primary care services. These benefits should not only be instituted explicitly and regularly revised, but also widely communicated to scheme beneficiaries who may not be used to seeking care at the primary level.

 A second obstacle identified is the lack of performance monitoring and health information systems. This challenge is particularly pervasive for primary care, as PHC facilities are more likely to be underfunded and lack sophisticated information technology systems. Even when data is gathered on PHC provider behaviour, such as in the Philippines, this may not be supported by strong digital systems and/or may not be linked to further purchasing decisions. A concrete example of data-driven monitoring influencing purchasing decisions can be found in Thailand’s NHSO, which collects micro-level claims data across all UCS providers and uses this to regularly revise capitation rates, benefits packages and provider contracts. This pathway from data collection to analysis to purchasing decisions represents an operational model that other countries in the region are aspiring toward. Thailand’s two other major purchasers, the SSO and the CGD, also lack the capacity for data-driven purchasing decisions, illustrating how even a high-performing strategic purchaser such as the NHSO may be limited when fragmentation or lack of coordination among different purchasers persists. In terms of health information systems, Indonesia stands out for its powerful P-Care application, which is directly linked to BPJS-K’s claims system. It allows near real-time tracking of services delivered by both public and private PHC providers. BPJS-K can monitor service volumes, diagnoses and treatment patterns on a daily basis, and the system is widely accessible, including via mobile devices. P-Care also facilitates referrals and captures key patient information, making it a central tool for data-informed purchasing. As strategic purchasing is the *evidence-based *allocation of resources for health, data and information systems are a key pre-requisite: health systems must shift entirely from paper-based to digital data collection mechanisms; have interoperable data systems, preferably aligned with international data standards^78^; and mandate collection of appropriate data points on patient outcomes, quality of care and costs to facilitate performance monitoring and planning.^79,80^ However, to support strategic purchasing, investments are needed not only in digital platforms but also in institutional capacity to collect and analyse data; an aim which most Southeast Asian countries are still striving toward.

 Finally, challenges remain in defining the role of private providers. This consideration applies to all levels of care (not only primary) and introduces questions on accreditation, contracting arrangements, provider payment rates and more. The private healthcare sector is a significant and growing force in Southeast Asia, but limited governance capacity among governments/purchasers may prevent the private sector from being leveraged for public health objectives. Public-private collaboration may take place on regional or provincial levels but not nationally—for example, with private providers being contracted by LGU health networks in the Philippines or CUP in Thailand. The Mitra Plumbon Healthcare Group in West Java exemplifies successful local collaboration by strategically expanding into second-tier cities to serve high volumes of JKN participants.^81^ This model leverages integrated digital systems and primary-first referral pathways to align private sector growth with public health equity and financial sustainability. Once again, capacity-building and knowledge transfer among ASEAN countries could illustrate possible models of public-private collaboration in health.

###  Policy Recommendations

 Our analysis has identified a series of challenges, opportunities and potential policy levers for strategic purchasing across four Southeast Asian countries. Based on this analysis, we have produced a set of clear, actionable and high-impact policy recommendations summarised in Table 4. The recommendations are grouped according to five broad themes: (1) governance, (2) financing, (3) PHC, (4) provider payment, and (5) private sector engagement.

**Table 4:** Summary of Policy Recommendations

**Theme**	**Recommendations**	**Relevant Countries**
Governance	Strengthen regional collaboration and knowledge transfer on strategic purchasing. Leverage existing ASEAN health networks to allow the sharing of case studies and lessons between countries.	All
Governance	Establish clear governance frameworks to grant purchasing agencies sufficient mandate, autonomy, purchasing power, and accountability mechanisms to implement strategic purchasing.	Vietnam, Philippines
Governance	Align public financial management rules with health system objectives to enable outcomes-based and performance-linked spending.	All
Financing	Align PHC budget allocations with population health needs, using data on utilisation, epidemiological profiles, and provider performance to guide resource allocation.	All
Financing	Reduce fragmentation in health financing schemes to enhance purchasing power, improve efficiency and promote equity in access to PHC benefits.	All
Financing	Invest in digital health information systems and institutional capacity to collect, analyse and use data to guide purchasing and resource allocation decisions.	All; Philippines and Vietnam in particular
PHC	Reinforce PHC as the foundation of UHC by implementing referral and gatekeeping mechanisms, and designing benefit packages centred on primary care.	Philippines and Vietnam
PHC	Invest in the supply-side provision of primary care, including workforce development and essential medicines, especially in rural and hard-to-reach areas.	Indonesia, Philippines, Vietnam
PHC	Communicate benefits packages widely to populations to increase PHC care-seeking and reduce unnecessary bypassing to specialist care.	Philippines, Vietnam
Provider payment	Move toward capitation and performance-based payment mechanisms for PHC, as appropriate, with carefully designed and regularly revised formulas. Draw on Thailand's QOF experience and other global expertise.	Indonesia, Philippines, Vietnam
Provider payment	Pilot performance-based financing initiatives before scaling, with robust monitoring and evaluation to identify successful models in-country.	Indonesia, Philippines
Provider payment	Ensure timely and predictable disbursement of payments to PHC providers, with adequate payment schedules and well-calculated rates that cover facility costs and avoid disincentivising high-quality care.	All; Vietnam and Indonesia in particular
Private sector engagement	Develop clearer accreditation, contracting and payment standards for private PHC providers to leverage the private sector for public health objectives.	Indonesia, Philippines

Abbreviations: PHC, primary healthcare; ASEAN, Association of Southeast Asian Nations; UHC, universal health coverage; QOF, quality and outcome framework.

###  Strengths and Limitations

 This study’s strengths lie in its policy-relevant and timely nature, drawing on a comprehensive literature review combined with in-depth expert stakeholder interviews. The use of the Strategic Health Purchasing Progress Tracking Framework as an analytical lens ensures systematic comparability across four diverse health systems (both within the study and with other health systems that have applied the Framework). Our study also benefits from the involvement of country-based researchers with direct knowledge of each health system, and health system leaders as expert interviewers to ensure policy relevance of the results. Finally, by examining four countries at different stages of implementing strategic purchasing, our study offers a comparative perspective grounded within Southeast Asia which is rare within health financing literature.

 Nonetheless, the present study has several limitations that should be acknowledged. Firstly, the evidence base is uneven across the four countries. Thailand has a comparatively rich body of published literature on strategic purchasing, whereas evidence on Indonesia, the Philippines and Vietnam relies more heavily on grey literature and policy documents. Secondly, the qualitative data gathered through interviews was drawn primarily from policy insiders, including national policy-makers, purchasing agency representatives and health financing experts. Whilst this ensured access to detailed institutional knowledge, the sample size of only 14 interviews across four countries was limited and may not fully represent the diversity of subnational experiences—particularly in large, decentralised systems such as Indonesia and the Philippines. Finally, as our study was intended as a high-level comparative policy analysis, it was not possible to examine in depth the equity implications of strategic purchasing arrangements for vulnerable groups, including informal workers, women, rural populations, migrants, and refugees. The extent to which purchasing reforms have reached these populations, and whether they have successfully narrowed health inequities, remains an important area for future research.

## Conclusion

 This multi-country study finds that Indonesia, the Philippines, Thailand, and Vietnam have all made deliberate progress toward UHC and health financing reform in recent years. Whilst challenges remain, particularly in incentivising PHC utilisation and raising standards of care, all four countries are trending toward enhanced strategic purchasing. This is being achieved through performance-based payment pilots, experiments with diverse provider payment methods and explicit benefits specification, among other functions.

 Whilst this study has focused on four relatively mature NHI systems, the findings carry broader relevance. For any LMICs with emerging or nascent health financing systems—where scheme coverage remains low, pooling is fragmented, and purchaser capacity is limited—the recent experiences of Indonesia and the Philippines may be particularly instructive. Both countries have made meaningful progress in strategic purchasing in recent years, without yet having achieved full system maturity as compared with Thailand’s NHSO. For tax-financed systems, where a purchaser-provider split may be absent or poorly defined, the key principles considered in this study are also pertinent. These include functioning Health Information Systems and data analysis capabilities, governance and accountability frameworks, well-considered and regularly revised provider payment mechanisms and crucially, the practice of using available evidence to guide decisions in purchasing and resource allocation.

 Overall, given persistent resource and fiscal space constraints in ASEAN, enhancing strategic purchasing of health services provides an opportunity to advance the region’s UHC goals. Government stewardship backed by strong political will, capacity-building and cooperation with (and among) national purchasers will all be required to achieve more strategic purchasing. There is also a need for enhanced data collection and crucially, for data to be analysed to guide resource allocation decisions. Whether achieved through government budgets, social health protection schemes or a mix of the two, these steps will pave the way for UHC; not only by making purchasing more strategic, but also by giving rise to well-governed, sustainable, integrated, and data-driven health systems with efficient uses of limited resources.

 Finally, however, it is important to recognise that strategic purchasing is one component of a complex system of global health. Even well-designed purchasing systems operate within, and are constrained by, broader systemic barriers. These include fiscal deficits and constrained domestic health budgets, particularly in the context of declining donor aid; governance fragmentation and limited institutional capacity and resources to implement health system reforms; the growing role of the private sector and the financialisation of health; and social determinants of health—including poverty, food insecurity, inadequate housing, and gender inequality—shape population health needs in ways that purchasing reforms cannot fully address.At the global level, geopolitical instability and the lingering effects of the COVID-19 pandemic are placing additional pressure on already constrained health budgets whilst also directly impacting population health. In this context, strategic purchasing should be understood as a necessary but not sufficient condition for UHC. Its impact will be greatest when pursued alongside broader investments in social protection, poverty reduction, health systems strengthening, and good governance.

## Acknowledgements

 We would like to acknowledge Dr. Jeremy Lim and Dr. Swee Kheng Khor for their early contributions to this research.

## Disclosure of artificial intelligence (AI) use

 Not applicable.

## Ethical issues

 Ethical approval for this research was granted by the National University of Singapore Institutional Review Board (NUS-IRB-2022-630).

## Conflicts of interest

 Authors declare that they have no conflicts of interest.

## Supplementary files


Supplementary file 1. Strategic Purchasing for Primary Healthcare in Indonesia.

Supplementary file 2. Strategic Purchasing for Primary Healthcare in the Philippines.

Supplementary file 3. Strategic Purchasing for Primary Healthcare in Thailand.

Supplementary file 4. Strategic Purchasing for Primary Healthcare in Vietnam.
