# Supplementary material for: Advancing Strategic Health Purchasing for Primary Healthcare in Southeast Asia: A Review of Policy and Practice in Indonesia, the Philippines, Thailand, and Vietnam
Source: Int J Health Policy Manag. 2026 Jul 28;15:9326. doi: 10.34172/ijhpm.9326 (PMC13770240; doi:10.34172/ijhpm.9326)
Supplement: Supplementary file 1 [file ijhpm-15-9326-s001.pdf]

Article title: Advancing Strategic Health Purchasing for Primary Healthcare in Southeast Asia: A Review of Policy and Practice in Indonesia, the Philippines, Thailand, and Vietnam

Journal name: International Journal of Health Policy and Management (IJHPM)

Authors' information: Capucine Barcellona[1]*, Michael Caampued[2], Shita Dewi[3], Jeremy Turner-Gorospe[1], Theepakorn Jithitikulchai[4], Nguyen Khanh Phuong[5], Nguyen Thu Ha[6], Ryan Rachmad Nugraha[7], Ong The Due[5], Gugma Vidal[1], Pacharee Wattanakulchai[4], Kiesha Prem[1]

[1]Saw Swee Hock School of Public Health, National University of Singapore, Singapore, Singapore.

[2]Philippine Society of Public Health Physicians, Manila, Philippines.

[3]Centre for Health Policy and Management, Faculty of Medicine, Public Health and Nursing, Universitas Gadjah Mada, Yogyakarta, Indonesia.

[4]Faculty of Economics, Thammasat University, Bangkok, Thailand.

[5]Health Strategy and Policy Institute, Ministry of Health, Hanoi, Vietnam.

[6]Department of Health Policy and Economics, Hanoi University of Public Health, Hanoi, Vietnam.

[7]Department of Family and Community Medicine, Faculty of Medicine, Public Health and Nursing, Universitas Gadjah Mada, Yogyakarta, Indonesia.

***Correspondence to**: Capucine Barcellona; Email: capubarc@outlook.com

Citation: Barcellona C, Caampued M, Dewi S, et al. Advancing strategic health purchasing for primary healthcare in Southeast Asia: a review of policy and practice in Indonesia, the Philippines, Thailand, and Vietnam. Int J Health Policy Manag. 2026;15:9326. doi:10.34172/ijhpm.9326

Supplementary file 1. Strategic Purchasing for Primary Healthcare in Indonesia

Indonesia

Background: Indonesia is an upper-middle income country with a population of 275.5 million, making it the most populous country in Southeast Asia and the fourth most populous in the world. Health financing integration has historically been challenging due to the country's large population size, archipelagic geographic features and devolved health and governance systems. The Jaminan Kesehatan Nasional (JKN) single-payer health insurance scheme was introduced in 2014 to unify its fragmented health financing system. The programme consolidated over 300 risk pools into a single national scheme managed by BPJS Kesehatan (BPJS-K)—now the world's largest single-payer health insurance system, covering 98.18% of the population by mid 2024.[26]

Despite the JKN's success in pooling funding for 98% of the population, Indonesia's archipelagic geography and decentralised governance create structural frictions that impede effective integration. The dispersion of the population across thousands of islands creates logistical bottlenecks; moreover, while services are technically covered under the JKN, supply-side constraints often lead to unavailability of medications or specialist services in remote facilities, forcing patients to incur high OOP payments.[27] Decentralisation has led to large heterogeneity in local prioritisation, with district-level health budget allocations ranging from as low as 3% to more than 18%.[28, 29] While JKN has incentivised private sector expansion, this growth has concentrated in urban centers, further exacerbating the rural-urban equity gap in service delivery.[30]

Primary healthcare system: Public PHC in Indonesia is provided through over 10,000 community health centres (known as Puskesmas) and their networks (known as Puskesmas Pembantu/Pustu), as well as village-based health services (known as Posyandu or Poskesdes). Funding for curative primary care delivered at Puskesmas is routed through the JKN. Some private primary care clinics are also contracted by BPJS-K to provide services under the JKN. Additional funding is provided to public facilities from central and regional governments. This funding is largely intended for PHC infrastructure development, facility support, operational costs and public health activities. Village-based Posyandu and Poskesdes health services operate fully through village government funding.

Financial management: BPJS-K pools funds from multiple revenue sources, including mandatory contributions and government subsidies, into one single source account known as the *Dana Jaminan Sosial (DJS)*. As sole purchaser for the JKN, BPJS-K has full autonomy over this fund—part of which is directed toward Puskesmas. PHC institutions (including Puskesmas, Posyandu and others) receive additional funding from central and regional government channels (including village government), although this investment may not always match local needs. There are ongoing challenges relating to the JKN's financial sustainability: the "missing middle", comprised of informal workers who struggle to maintain active membership, creates a persistent cumulative deficit, as the single-payer scheme lacks a consistent revenue stream from this demographic.[31]

Benefits specification: Primary care benefits for the JKN scheme are explicitly defined. These are comprehensive, covering non-specialist care such as service administration; individual promotive

and preventive care; medical examinations, treatments and consultations; non-specialistic medical measurements, surgical or non-surgical; non-specialist dental care; pharmaceutical services, medical device and consumable medical materials; diagnostic examinations (laboratory test, imaging exam, etc.); family planning; routine immunisation; screenings; teleconsultations; and primary level inpatient care according to specific indications. Benefits specification is conducted by the Ministry of Health (MoH) through cost-effectiveness assessments, with revisions codified through Presidential Regulations and Amendments as required.[32]

Contracting arrangements: Public Puskesmas and private PHC clinics are required to undergo licensing, known as credentialing, through BPJS-K in order to be contracted as part of the JKN scheme. This credentialing uses predetermined metrics, with a particular focus on supply-side and process readiness. Moreover, to ensure the quality of clinical care, the MoH conducts quality of care monitoring known as “accreditation” in cooperation with various independent accreditation agencies. This MoH-supervised accreditation applies to both private and public primary care facilities (e.g., private laboratories, private clinics and Puskesmas). No explicit contracting is required for government-funded public health activities beyond the JKN benefits package, and these automatically involve public health facilities as needed. While public providers (Puskesmas) are not bound by explicit, performance-contingent contracts for non-JKN services, they must adhere to broader MoH service delivery regulations.

However, the absence of formal contracting creates significant operational and strategic hurdles. The lack of a clear demarcation between JKN-funded activities and MoH supply-side financing may limit BPJS-K’s ability to function as a strategic purchaser. Without explicit contracts defining obligations and expectations, the purchaser has limited leverage to enforce quality standards or efficiency gains.[33] Public facilities are mandated to provide services to all government-subsidized members, often without commensurate investments in infrastructure or staffing. This open-door mandate, in the absence of capacity-linked contracting, has led to severe overcrowding and prolonged waiting times.[34] Recent studies indicate that these barriers significantly diminish patient satisfaction and can delay essential treatments.[35,36] Consequently, while the automatic involvement of public providers ensures a baseline of access, it reinforces a passive purchasing model that struggles to reward high-performing facilities or address localised system bottlenecks.

Provider payment: Capitation payments are made to PHC providers on a monthly basis, with the exception of maternal and urgent inpatient care, which is funded on a retrospective line-item basis. Payment rates are determined by the MoH, with some differences between public and private facilities at the primary care level;[37] currently, capitation rates for private providers are higher than those for public care providers, and there are variations in allocation for different private providers. As of 2019, performance-based elements were introduced to this capitation funding based on criteria relating to 1) contact rate; 2) managed chronic care patient ratio; and 3) ratio of non-specialist referrals.[39]

Beyond the JKN, public providers receive an additional operating budget from the MoH on an annual basis. District Health Offices and Puskesmas jointly plan and submit funding requests for PHC services ("Rencana Kegiatan Anggaran") ahead of the annual budget cycle. Starting from 2022, the MoH established a special incentive for PHC known as the Insentif Upaya Kesehatan Masyarakat. Additional incentives are available based on claims from PHC centres offering inpatient services, such as birth deliveries or basic obstetric emergencies.[40]

Despite recent adjustments, several challenges persist in Indonesia's capitation model. Providers, especially in the private sector, report that current rates remain insufficient to recover the full costs of clinical personnel and medications.[31] Complex claims administration and procedures that exceed capitation caps have been found to distort provider behaviour, often incentivising unnecessary referrals to higher levels of care.[38] Current performance-based system may inadvertently exacerbate inequities; without adequate risk adjustment, the withholding of payments for failing to meet far-reaching targets often penalizes already under-resourced facilities in remote and rural areas.[41]

Performance monitoring: The JKN requires PHC providers to meet specific monthly targets to attain full disbursement of performance-based capitation payments (including contact rate, managed chronic care patient ratio and ratio of non-specialist referrals, as described above). Independent Cost and Quality Control committees (Tim Kendali Mutu dan Kendali Biaya, or TKMKB) are based at the national, provincial and BPJSK branch-level to monitor health service quality. In primary care, they conduct ad-hoc utilisation reviews and audits of non-specialist referral ratios using TACC criteria (Time, Age, Complication and Comorbidity). The extent to which this informs PHC purchasing decisions is unknown. The TKMKB's "cost control" function

is more evident in specialist and hospital care, where the appropriateness of claims and reimbursements are reviewed to prevent fraud. Nonetheless, there are variations in the practice of the TKMKB from province to province. BPJS-K also uses an information system application, P-Care, that is used to monitor provider behaviour (public and private) and is linked directly to provider claims. This fragmentation is compounded by data management issues, where unsynchronised household databases make it difficult to identify and validate poor households for subsidies.

At the health system level, an integrated reporting platform known as ASDK (Aplikasi Satu Data Kesehatan—Health Single Data Application) gathers performance indicators from Puskesmas nationwide. A national integrated digital health platform, SATUSEHAT, has also been developed to facilitate health information exchange but is not currently not used for performance monitoring. Performance monitoring for public health priority programmes beyond the JKN (e.g., HIV/AIDS and TB screening) has been conducted regularly by the MoH through different vertical working units. Integration of these vertical information monitoring systems into national platforms is currently underway, and there is a recognition of the importance of information systems to support strategic purchasing.
