# Supplementary material for: Advancing Strategic Health Purchasing for Primary Healthcare in Southeast Asia: A Review of Policy and Practice in Indonesia, the Philippines, Thailand, and Vietnam
Source: Int J Health Policy Manag. 2026 Jul 28;15:9326. doi: 10.34172/ijhpm.9326 (PMC13770240; doi:10.34172/ijhpm.9326)
Supplement: Supplementary file 2 [file ijhpm-15-9326-s002.pdf]

Article title: Advancing Strategic Health Purchasing for Primary Healthcare in Southeast Asia: A Review of Policy and Practice in Indonesia, the Philippines, Thailand, and Vietnam

Journal name: International Journal of Health Policy and Management (IJHPM)

Authors' information: Capucine Barcellona[1]*, Michael Caampued[2], Shita Dewi[3], Jeremy Turner-Gorospe[1], Theepakorn Jithitikulchai[4], Nguyen Khanh Phuong[5], Nguyen Thu Ha[6], Ryan Rachmad Nugraha[7], Ong The Due[5], Gugma Vidal[1], Pacharee Wattanakulchai[4], Kiesha Prem[1]

[1]Saw Swee Hock School of Public Health, National University of Singapore, Singapore, Singapore.

[2]Philippine Society of Public Health Physicians, Manila, Philippines.

[3]Centre for Health Policy and Management, Faculty of Medicine, Public Health and Nursing, Universitas Gadjah Mada, Yogyakarta, Indonesia.

[4]Faculty of Economics, Thammasat University, Bangkok, Thailand.

[5]Health Strategy and Policy Institute, Ministry of Health, Hanoi, Vietnam.

[6]Department of Health Policy and Economics, Hanoi University of Public Health, Hanoi, Vietnam.

[7]Department of Family and Community Medicine, Faculty of Medicine, Public Health and Nursing, Universitas Gadjah Mada, Yogyakarta, Indonesia.

***Correspondence to**: Capucine Barcellona; Email: capubarc@outlook.com

Citation: Barcellona C, Caampued M, Dewi S, et al. Advancing strategic health purchasing for primary healthcare in Southeast Asia: a review of policy and practice in Indonesia, the Philippines, Thailand, and Vietnam. Int J Health Policy Manag. 2026;15:9326. doi:10.34172/ijhpm.9326

Supplementary file 2. Strategic Purchasing for Primary Healthcare in the Philippines

The Philippines

Background: The Philippines is another lower-middle income archipelagic country in Southeast Asia, comprising over 7,000 islands and islets and featuring a highly devolved health system. Health was enshrined as a constitutional right in 1987, and the landmark Universal Health Coverage Law passed in 2019 paved the way for system-wide reforms.[42] Nearly the entire population (115.56 million individuals) is enrolled under the National Health Insurance (NHI) programme implemented by the Philippine Health Insurance Corporation (PhilHealth), established in 1995, which operates alongside devolved Local Government Units (LGUs) responsible for frontline service delivery.

Primary healthcare system: PHC in the Philippines is largely the responsibility of Local Government Units (LGUs). There are 120 primary-level LGUs in the country, with each covering a population between 18,800 and 4,344,829. There is therefore great variation in the population size managed by each LGU. The Local Government Code of 1991 charges LGUs to manage Rural Health Units (RHUs) and barangay health stations to deliver PHC services.[43] These public facilities receive LGU and Department of Health (DoH) funding to cover most operational costs. As RHUs and barangay health stations fall under the jurisdiction of individual LGUs, the availability of funds is dependent on each LGU's internal priorities.

In 2020, PhilHealth introduced further expansions to primary care benefit delivery through the Konsultasyong Sulit at Tama (Konsulta) programme, which integrated prior models of Primary Care Benefits packages. This integration effectively increased services and augmented financing for accredited Primary Care Facilities to support delivery. Konsulta contracts public as well as accredited private clinics, including for e-consultations. Beyond LGU and Konsulta funding, some PHC services are provided through the Sustainable Development Goals (SDG) Benefits package—including family planning, maternity care, animal bites and treatment for malaria, HIV/AIDS and TB. However, many patients continue to bypass PHC in favour of direct access to hospital-based care, undermining efficiency and cost-effectiveness.

Financial management: Duplicative allocation streams exist within the Philippine health financing systems. LGUs have devolved functions to own health facilities and deliver healthcare, the DoH continue provisions for human resource, facility investments, program commodities (i.e. vaccinations) and health system development assistance to LGUs—in part, with the aim of mitigating the variable financial capacities of LGUs. These are often determined by equity-based allocations that have historically defined the DoH's approach to LGU stewardship and assistance. LGU-level funds are then aggregated through various sources, including Internal Revenue Allotment local revenues, DoH funds, PhilHealth payments, grants and donor loans.[44] Attempts have been made to correct devolved financing roles through the Mandanas-Garcia ruling instigating the formulation of the DoH Devolution Transition Plan (DTP) 2022-2024.[45,46] Guidance was provided to LGUs reformulate investment plans to align with the DoH's DTP, as the DoH aims to ease some of its roles in allocating for LGU-directed commodities and other resources. This, however, was been fully implemented.

Whilst PhilHealth enjoys greater flexibility in its financial management as compared to the DoH, its functions are mostly directed toward specialist care with the exception of the relatively small-scale Konsulta package. PhilHealth has the authority to redirect further funds toward PHC; efforts are underway to encourage greater integration and a primary care focus through the NHI.

Benefits specification: LGUs do not define explicit benefits packages for public primary care services under their jurisdiction. However, according to the Local Government Code and the UHC Law, LGUs are mandated to oversee primary care by delivering population-based services and managing local health networks.

PhilHealth's Konsulta PHC package has defined benefits including initial and follow-up consultations, health screenings and assessments, 13 diagnostic services and 21 drugs and medicines which are all free at the point of care. While these benefits were initially only available for low-income beneficiaries and Overseas Filipino Workers (OFW), access has since been expanded to all Filipinos.

Contracting arrangements: LGU primary healthcare networks borrow their accreditation requirements from PhilHealth, and include an operational assessment of health offices, facilities, services, human resources and other. Private providers may participate in LGU networks through formal contractual arrangements with a province- or city-wide health system; this is generally done for laboratory works, diagnostics and therapies. For PhilHealth's Konsulta package, only non-hospital facilities are eligible for accreditation; this includes rural health units, private clinics, outpatient departments, company clinics and school clinics. Application is made through a local health insurance office, with 2548 Konsulta providers accredited as of May 2024.

The Philippines has encountered some challenges in the contracting of private providers. The system of accreditation has not fully prevented quality of care concerns, and enforcement and monitoring remain uneven. Global evidence suggests that accreditation improves minimum standards and service capability,[47] yet country gaps in inspection capacity, weak mechanisms for the de-accreditation of poor performers and workforce and financing issues mean that quality is not consistently assured across contracted private facilities. Moreover, the contracting of private providers has largely been a per-facility engagement.[48] This points to systemic health reform issues and complex administrative bottlenecks. Complex administrative and legal requirements for contracting, coupled with a lack of legal precedents to integrate private providers into mixed

public-private contracting networks, limit the private sector's participation in the implementation of UHC reforms.

Provider payment: LGUs generally provide input-based, line-item budgets on an annual basis to public PHC providers under their jurisdiction. Selective contracts with private providers may involve a range of payment methods, according to explicit contracting agreements. Annual capitation funds are released in advance to healthcare providers delivering Konsulta, using per capita rates of PHP1,700 (~USD 30) for all providers, up from earlier rates of PHP500 for public facilities and PHP750 for private facilities.[49,50]

Performance monitoring: LGUs are monitored by the DoH through an LGU scorecard measuring a range of indicators, including each LGU's success in implementing primary care within their province. In turn, LGUs are required to monitor the performance of their networks through annual audit mechanisms; however, the extent to which these audits are carried out is unclear. Much like other services delivered by PhilHealth, Konsulta services are subject to regular surveillance, audits and rating according to set standards. Nonetheless, systems-level performance monitoring by PhilHealth is limited. This is particularly true for primary care, as Konsulta does not include all PHC facilities nor the entire Filipino population at this stage.

In 2023, PhilHealth implemented network contracting arrangements to test integrative local health system reforms through the PCPN (Primary Care Provider Network) Sandbox.[51] This was piloted in four local governments (three provinces, one city) with the objective of improving utilisation and delivery and establishing efficiencies in financing through a pooled Special Health Fund. The Sandbox implementation involved regular audits, monitoring and evaluation—providing PhilHealth an opportunity to learn from diverse delivery models. Over 2024-2025, a more expansive network contracting and benefit delivery model termed "Health Care Provider Networks" (HCPN) is being rolled out to 49 LGUs, including the 4 PCPN Sandbox sites. This shift expands prospective payments to networks beyond primary care, including select hospital-based conditions and outpatient drug benefits.
