# Supplementary material for: Advancing Strategic Health Purchasing for Primary Healthcare in Southeast Asia: A Review of Policy and Practice in Indonesia, the Philippines, Thailand, and Vietnam
Source: Int J Health Policy Manag. 2026 Jul 28;15:9326. doi: 10.34172/ijhpm.9326 (PMC13770240; doi:10.34172/ijhpm.9326)
Supplement: Supplementary file 3 [file ijhpm-15-9326-s003.pdf]

Article title: Advancing Strategic Health Purchasing for Primary Healthcare in Southeast Asia: A Review of Policy and Practice in Indonesia, the Philippines, Thailand, and Vietnam

Journal name: International Journal of Health Policy and Management (IJHPM)

Authors' information: Capucine Barcellona[1]*, Michael Caampued[2], Shita Dewi[3], Jeremy Turner-Gorospe[1], Theepakorn Jithitikulchai[4], Nguyen Khanh Phuong[5], Nguyen Thu Ha[6], Ryan Rachmad Nugraha[7], Ong The Due[5], Gugma Vidal[1], Pacharee Wattanakulchai[4], Kiesha Prem[1]

[1]Saw Swee Hock School of Public Health, National University of Singapore, Singapore, Singapore.

[2]Philippine Society of Public Health Physicians, Manila, Philippines.

[3]Centre for Health Policy and Management, Faculty of Medicine, Public Health and Nursing, Universitas Gadjah Mada, Yogyakarta, Indonesia.

[4]Faculty of Economics, Thammasat University, Bangkok, Thailand.

[5]Health Strategy and Policy Institute, Ministry of Health, Hanoi, Vietnam.

[6]Department of Health Policy and Economics, Hanoi University of Public Health, Hanoi, Vietnam.

[7]Department of Family and Community Medicine, Faculty of Medicine, Public Health and Nursing, Universitas Gadjah Mada, Yogyakarta, Indonesia.

***Correspondence to**: Capucine Barcellona; Email: capubarc@outlook.com

Citation: Barcellona C, Caampued M, Dewi S, et al. Advancing strategic health purchasing for primary healthcare in Southeast Asia: a review of policy and practice in Indonesia, the Philippines, Thailand, and Vietnam. Int J Health Policy Manag. 2026;15:9326. doi:10.34172/ijhpm.9326

Supplementary file 3. Strategic Purchasing for Primary Healthcare in Thailand

Thailand

Background: Thailand is an upper-middle income country globally renowned for its commitment to UHC and transformative journey toward health system improvements. Three complementary health financing schemes exist in Thailand: 1) the Social Security Scheme (SSS) covering private employees (financed through state subsidies and employer/employee contributions); 2) the Civil Servant Medical Benefit Scheme (CSMBS) covering civil servants and their close family (financed through a tax-based, non-contributory government budget); and 3) the Universal Coverage Scheme (UCS), targeting all individuals not covered under the SSS or CSMBS. The UCS is Thailand's largest health financing scheme—covering 49.8 million people, or 74% of the population—and is financed through a tax-based government budget.[52]

Primary healthcare system: Thailand's PHC system is operated by the Ministry of Public Health (MoPH) and acts as the first point of contact for patients thanks to mandatory referral systems. The nationwide PHC system functions through a local network of primary care facilities including health centres and community hospitals (District Health Systems, DHS). Community-based healthcare is well-developed, with a strong focus on health promotion and prevention and engagement of community health volunteers. Whilst beneficiaries of the UCS scheme primarily obtain primary care at public facilities (such as DHS), the SSS and CSMBS schemes can cover PHC services delivered by private providers, subject to certain preconditions.

Financial Management: The National Health Security Office (NHSO) plays a central role in the financial management of primary care for UCS beneficiaries, as well as promotive/preventive health for the entire Thai population. Each year, the government—through the Ministry of Finance (MoF) and the Cabinet—allocates a budget based on a proposal submitted by the NHSO. Once approved by Parliament, this budget is managed autonomously by the NHSO. Similarly, the Social Security Office (SSO) under the Ministry of Labour (MoL) administers the SSS, with responsibilities for collecting contributions and managing the scheme's funds. In contrast, the Comptroller General Department (CGD), which acts as the managing agency of the CSMBS, does not have a formal legal mandate to function as a purchaser. It nonetheless plays a key role in budget formulation and execution, working in collaboration with the MoF, regional health offices and healthcare providers.

Benefits specification: The UCS has a comprehensive PHC benefits package, explicitly defined in the National Health Security Act of 2002.[53] Regular revisions to the benefits package are made by the NHSO, the purchaser for the UCS, with the assistance of Health Technology Assessment processes—notably through Thailand's Health Intervention and Technology Assessment Program (HITAP) Foundation. UCS benefits were upgraded in 2023 to include free items for elderly beneficiaries (e.g., eyeglasses, dentures, adult diapers).[55] The SSS has a similar benefits package defined in the Social Security Act of 1990.[54] Revisions take place periodically as needed, depending on population health needs and policy changes. Whilst PHC services at public hospitals are fully covered, a price cap of 2,000 baht per month is set on private outpatient care. Finally, the CSMBS also includes comprehensive PHC benefits; these are set annually by a technical advisory committee consisting of medical experts and approved by the CGD.

As the SSS and CSMBS schemes are primarily designed for curative care, the NHSO (managing agent of the UCS) oversees primary-level prevention and promotion activities for members of all schemes (i.e., for all Thai citizens).

Contracting arrangements: As part of the UCS, NHSO contracts both public providers (mainly DHS) and private providers; for primary care, this is done through the Contracting Unit for Primary Care (CUP). Whereas public providers are automatically enrolled, private providers must meet specific accreditation and quality standards. All UCS facilities sign contracts including detailed provisions on service delivery requirements, quality standards and performance monitoring, among other features.

As the SSS contracts mainly with large hospitals, primary care clinics (both public and private) operate as subcontractors of these hospitals. These large hospitals and PHC clinics may be public or private and are contracted on a competitive basis through the Social Security Office's provincial offices. Conversely, the CSMBS scheme does not directly contract providers but simply reimburses claims to users or healthcare institutions with conditions on price ceilings and based on the National List of Essential Medicines.

Provider payment: The UCS pays DHS and its other primary care providers through capitation payments based on explicit formulas. These formulas consider utilisation rates and unit costs, among other data. Performance incentives are also provided under a Quality and Outcome Framework (QOF) intended to reward quality care and patient satisfaction. The SSS also uses capitation payments for PHC, with rates set by the SSO based on patient demographics, healthcare costs and clinical outcomes data.

As primary care services under the CSMBS are reimbursed directly to providers by the CGD, payment is made on a fee-for-service basis with a ceiling. Claims are submitted by providers and later reviewed, verified and reimbursed by the CGD. Currently, the expenditure per capita of the CSMBS surpasses that of the other two schemes, reaching a value three times greater than the UCS' per capita expenditure. This is attributable to the relatively generous benefits package available to civil servant beneficiaries, combined with the predominance of fee-for-service payment for outpatient care under the CSMBS which creates incentives for higher service utilisation and contributes to cost escalation.

Performance monitoring: The NHSO conducts performance monitoring functions for the UCS. Microdata on services provided and reimbursements made is collected and analysed in light of broader health system objectives. This information is used to inform policy-making decisions and to guide future purchasing practices. Specific indicators are laid out for PHC, including Health Information Systems (HIS); health promotion activities; disease prevention; home visits to vulnerable groups; laboratory diagnostic services; and care for patients at risk of diabetes/hypertension.

Thailand's two other main purchasers—the SSO for the SSS and especially, the CGD for the CSMBS—lack advanced performance monitoring functions. Whilst the SSO conducts regular assessments and quality control audits, there are no accountability frameworks or performance monitoring procedures for PHC. Similarly, while the CGD monitors the general performance of the CSMBS, there is limited data collection and no use of this data to guide purchasing practices.
