# Supplementary material for: Advancing Strategic Health Purchasing for Primary Healthcare in Southeast Asia: A Review of Policy and Practice in Indonesia, the Philippines, Thailand, and Vietnam
Source: Int J Health Policy Manag. 2026 Jul 28;15:9326. doi: 10.34172/ijhpm.9326 (PMC13770240; doi:10.34172/ijhpm.9326)
Supplement: Supplementary file 4 [file ijhpm-15-9326-s004.pdf]

Article title: Advancing Strategic Health Purchasing for Primary Healthcare in Southeast Asia: A Review of Policy and Practice in Indonesia, the Philippines, Thailand, and Vietnam

Journal name: International Journal of Health Policy and Management (IJHPM)

Authors' information: Capucine Barcellona[1]*, Michael Caampued[2], Shita Dewi[3], Jeremy Turner-Gorospe[1], Theepakorn Jithitikulchai[4], Nguyen Khanh Phuong[5], Nguyen Thu Ha[6], Ryan Rachmad Nugraha[7], Ong The Due[5], Gugma Vidal[1], Pacharee Wattanakulchai[4], Kiesha Prem[1]

[1]Saw Swee Hock School of Public Health, National University of Singapore, Singapore, Singapore.

[2]Philippine Society of Public Health Physicians, Manila, Philippines.

[3]Centre for Health Policy and Management, Faculty of Medicine, Public Health and Nursing, Universitas Gadjah Mada, Yogyakarta, Indonesia.

[4]Faculty of Economics, Thammasat University, Bangkok, Thailand.

[5]Health Strategy and Policy Institute, Ministry of Health, Hanoi, Vietnam.

[6]Department of Health Policy and Economics, Hanoi University of Public Health, Hanoi, Vietnam.

[7]Department of Family and Community Medicine, Faculty of Medicine, Public Health and Nursing, Universitas Gadjah Mada, Yogyakarta, Indonesia.

***Correspondence to**: Capucine Barcellona; Email: capubarc@outlook.com

Citation: Barcellona C, Caampued M, Dewi S, et al. Advancing strategic health purchasing for primary healthcare in Southeast Asia: a review of policy and practice in Indonesia, the Philippines, Thailand, and Vietnam. Int J Health Policy Manag. 2026;15:9326. doi:10.34172/ijhpm.9326

Supplementary file 4. Strategic Purchasing for Primary Healthcare in Vietnam

Vietnam

Background: Vietnam is a lower-middle income country with a population of 98.9 million and a mix of public and private healthcare facilities, despite its socialist market orientation. The country's first Social Health Insurance law was passed in 2008, but out-of-pocket payments remain high. The fiscal sustainability of the health system is challenged due to an ageing population and an increasing burden of Non-Communicable Diseases (NCDs).[56,57] Currently, over 93% of Vietnam's population is covered by the Social Health Insurance (SHI) Fund. The SHI is the country's single social health protection scheme and collects revenue from a range of sources (including employers/employees, individual households and government subsidies). The fund holder for the SHI is Vietnam Social Security (VSS), although the MoH is responsible for key

purchasing questions including which services to be purchased, how to purchase them and at which prices. Whereas curative services are provided through the SHI, preventive care (including vaccinations, health promotion and family planning at the PHC level) is financed through central and local government budgets.

Primary healthcare system: Vietnam's healthcare system is divided into four levels: central, provincial, district and commune. Until 2024, curative and preventive PHC services are provided at approximately 11,100 Commune Health Centres (CHC), with each CHC typically staffed by 5 personnel including one general doctor in 87.7% of cases.[58,59] The size of these communes varied widely, generally falling between 2,000-12,000 inhabitants but with some large communes covering a population of over 100,000. As of July 2025—following Vietnam's administrative system reform, which removed district level authorities and merged provinces and communes nationwide—the total number of provinces has decreased from 63 to 34 and the number of CHCs has been reduced to only 3,321. In addition to CHCs, primary care is also available at public District Health Centres (DHCs) or at private facilities, many of which are now contracted under the SHI. While PHC services are generally free or low-cost, many individuals bypass CHC care in favour of specialist care directly.[60] This may be, in part, due to Vietnam's bypassing policy whereby patients can seek treatment at district and provincial hospitals without referrals from CHCs. Nonetheless, data from the VSS indicates that over 70% of covered outpatient visits occur at the primary care level, with 50% of these at the district level and 20% at the commune level.

Financial management: The VSS fund operates as a single fund, whereby provincially raised revenues are pooled centrally. Provincial Social Security offices, Provincial Departments of Health and Provincial People's Committees are jointly responsible for implementing SHI policies at the provincial level and ensuring the availability of funds to subsidise premiums for beneficiaries. For primary care, DHCs consolidate SHI claims on behalf of their subordinate CHCs and for their own service provision. For government-funded programmes outside of the SHI (i.e., preventive services) budgets are planned annually and channelled to providers via the MoH or provincial Departments of Health (DoH).

Benefits specification: The SHI has an explicitly defined benefits package for curative primary care at the commune level, inclusive of 76 basic services (e.g., first aid, blood tests, normal birth delivery) and 241 essential medicines. This package was defined in 2017 through a Ministry of

Health Circular and applies to CHCs and DHCs.[61] The list of SHI-reimbursable medicines is revised by the MoH every 2-3 years. Co-payment rates for SHI-covered benefits (including medicines, services and medical devices) range from 0 to 20%.[62]

Whilst there is no explicit benefits package for preventive primary care, MoH Circular No. 30 in 2024 lists essential prevention, promotion and primary care services to be funded by the state.[63] These cover eight basic services listed in Declaration of Alma-Ata, including health education, immunisation, family planning, communicable disease surveillance and more. DHCs and CHCs are mandated to carry out these functions. However, provinces are required to allocate their own budgets to finance these preventive health services—resulting in limited practical implementation.

Contracting arrangements: All public facilities receive state funding directly and therefore do not require contracting. PHC facilities receive their funding primarily from their Provincial DoH. In addition, DHCs currently operate under an "autonomy" mechanism whereby their dependence on state budget allocations is gradually being reduced. Not all commune-level CHCs provide SHI-covered services, with some only delivering preventive healthcare. For public PHC providers which *do* deliver SHI-funded services, annual contracts with the VSS are required, serving as the basis for SHI fund allocations. However, only DHCs sign contracts with the VSS, as CHCs are subordinate units and cannot contract directly: CHC claim-seeking is conducted by DHCs on behalf of their subordinate CHCs. The SHI can also contract private clinics to provide PHC services, though accreditation processes are not well-defined or sufficiently selective.[5]

Provider payment: To carry out preventive health activities, public facilities receive state subsidies using a mix of global budget and line-item mechanisms. For curative care, the SHI primarily pays PHC providers on a fee-for-service basis. Capitation was also used as a payment method for PHC between 2009 and 2021 but was discontinued. A novel capitation method was later implemented in July 2021 but abandoned in December of the same year.[62] It was noted that the COVID-19 pandemic caused significant irregularities in healthcare activities, deviating from the usual patterns that had originally been regulated. Ultimately, authorities argued that implementing the scheme under such conditions would not ensure adequate costs for healthcare facilities under the SHI scheme.[64,65] These factors, coupled with the relative simplicity of the existing fee-for-service model, explain why fee-for-service remains the primary payment method for both inpatient and outpatient services. In March 2026, the Vietnam Ministry of Health

convened a high-level workshop to initiate the development of a Diagnosis-Related Group (DRG) payment method, focusing on establishing clinical coding and data standards while outlining a framework for future implementation.

Performance monitoring: The MoH regularly releases monitoring and evaluation indicators for the country's health system. Some of these indicators are related to primary care, such as the percentage of CHCs implementing preventive and non-communicable disease management (%); the percentage of children under 1 year old fully vaccinated; etc. CHCs do not report on these indicators directly but rather submit reports to higher-level units (e.g., DHCs), which then share aggregate reports with the MoH. Beyond the SHI, CHCs and DHCs implement several priority public health programmes such as HIV/AIDS and TB care and report on requested indicators for these programmes to higher-level units. This MoH data, however, is not widely used to guide strategic purchasing decisions for curative or preventive care.
